# Curcumin Antagonizes Glucose Fluctuation-Induced Renal Injury by Inhibiting Aerobic Glycolysis via the miR-489/LDHA Pathway

**DOI:** 10.1155/2021/6104529

**Published:** 2021-08-18

**Authors:** Xiaomei Fu, Jianfang Zhang, Xuanjie Huang, Zhifeng Mo, Ziyang Sang, Wenfei Duan, Wenfeng Huang

**Affiliations:** ^1^Department of Pediatrics, The First Affiliated Hospital of Henan University, Kaifeng, Henan, China; ^2^Department of Emergency and Disaster Medical Center, The Seventh Affiliated Hospital of Sun Yat-sen University, Shenzhen, China; ^3^Department of General Surgery, The First Affiliated Hospital of Henan University, Kaifeng, Henan, China

## Abstract

It has been considered that glucose fluctuation (GF) plays a role in renal injury and is related to diabetic nephropathy (DN) development. But the mechanism is still unclear. Aerobic glycolysis has become a topical issue in DN in recent years. There is an internal connection between GF, aerobic glycolysis, and DN. Curcumin (Cur) is a principal curcuminoid of turmeric and possesses specific protective properties in kidney functions. Cur also participates in the regulation of aerobic glycolysis switch. In this study, we first measured the levels of aerobic glycolysis and evaluated Cur's inhibitory ability in a cell model of HEK-293 under the condition of oscillating high glucose. The results indicated that GF exacerbated inflammation injury, oxidative stress, and apoptosis in HEK-293 cell, while Cur alleviated this cytotoxicity induced by GF. We found that GF increased aerobic glycolysis in HEK-293 cells and Cur presented a dose-dependent weakening effect to this exacerbation. Next, we built a panel of 17 miRNAs and 8 lncRNAs that were previously reported to mediate the Warburg effect. Our RT-qPCR results indicated that GF reduced the miR-489 content in the HEK-293 cell model and Cur could prevent this downregulation. Then, we planned to explore the character of miR-489 in Cur-triggered attenuation of the Warburg effect under GF condition. Our findings presented that Cur prevented GF-triggered aerobic glycolysis by upregulating miR-489 in HEK-293 cells. Next, we choose the miR-489/LDHA axis for further investigation. We confirmed that Cur prevented GF-triggered aerobic glycolysis via the miR-489/LDHA axis in HEK-293 cells. In conclusion, this study presented that Cur prevented GF-triggered renal injury by restraining aerobic glycolysis via the miR-489/LDHA axis in the HEK-293 cell model.

## 1. Introduction

Diabetic nephropathy (DN) is a typical diabetes mellitus- (DM-) related microvascular complication and one of the main causes of end-stage renal disease worldwide, which seriously endangers human health [1]. The latest report from World Health Organization indicated that there were approximate 463 million DM adults worldwide in 2014, and about 1/3 of them will develop DN [2]. It has been considered that glucose fluctuation (GF) is involved to exacerbation of renal injury in DM patients and is related with the department of DN. Short-dated glucose variability is related with the reduction of glomerular filtration rate and triggers an increased risk of chronic kidney disease [3]. GF condition had apoptosis-inducing and oxidative stress effects on different cells, including glomerular mesangial cells (MCs) [4], podocytes [5], and vascular endothelial cell [6]. However, the mechanism between GF and DN still remains unclear.

Mitochondria occupy a crucial role in cellular energy metabolism. Mitochondrial energy metabolism is a topical issue in DN in recent years, especially aerobic glycolysis (the “Warburg effect”). The flux of aerobic glycolysis, presented as glucose fermentation to lactate under sufficient levels of oxygen, was first found in cancer cells and regarded as one of the main hallmarks of cancer cells, leading to tumorigenesis and cancer cell rapid proliferation [7]. Interestingly, it was found that the upregulation of the Warburg effect deeply triggered the activation of myofibroblasts and affected both the number and function of podocytes [8, 9]. GF could deteriorate inflammation damage and apoptosis injury via intensifying the aerobic glycolysis in MC cell model [10]. These results have provided intrinsic relationships between GF, aerobic glycolysis, and DN, suggesting a potential association between the three.

Curcumin (Cur) is a principal curcuminoid of *Curcuma longa* (turmeric), making up around 77% of the total curcuminoids in the plant. Turmeric has been used in China for over millennia because of its medicinal properties and potential health benefits to metabolic disease [11]. Antioxidant and anti-inflammatory activities are regarded as two main benefits of Cur [12]. Cur possesses specific protective properties in kidney functions. Curcumin was found to inhibit podocyte cell apoptosis, accelerating cell autophagy, alleviating podocyte EMT, and reducing inflammation injury in the in vivo and in vitro models of DN [13–16]. A randomized, double-blind trial indicated Cur to be an effective treatment to ameliorate proteinuria in patients with type 2 DM [17]. Cur also takes part in the mediation of aerobic glycolysis. Cur could suppress cancer cell growth via regulating aerobic glycolysis, such as gastric tumor cell [18, 19], colon adenocarcinoma cell [20, 21], hepatocellular carcinoma cell [22, 23], and breast epithelial cells [24]. Thus, taken into consideration that Cur presents the ability of kidney protection and property of aerobic glycolysis mediation, it is meaningful to investigate whether Cur alleviates GF-induced renal injury via regulating aerobic glycolysis.

Noncoding RNAs, including microRNA (miRNA) and long noncoding RNA (lncRNA), are involved in numerous biological actions [25]. Both miRNA and lncRNA have been deeply studied in the fields of regulatory mechanism of aerobic glycolysis. For example, the miR-455-3p/FOXM1 axis increased lactate production, glucose uptake, and ATP generation in two human lung cancer cells (H1299 and A549) [26]. It indicated that the miR-186-3p/EREG axis triggered aerobic glycolysis to orchestrate tamoxifen resistance in breast cancer of ER-positive [27]. More than that, there are a host of miRNAs and lncRNAs involving in regulation of aerobic glycolysis, such as miR-142-3p [28], lncRNA-KCNQ1OT1 [29], and LINC01123 [30].

In this study, we first measured the levels of aerobic glycolysis and evaluated Cur's protective effects in HEK-293 cells under the condition of oscillating high glucose. After that, we built a panel of 17 miRNAs [26–29, 31–43] and 8 lncRNAs [29, 30, 32, 35, 36, 43–45] that were previously reported to be regulators of the Warburg effect. After a range of experiments, we found miR-489/LDHA as vital modulator axis in kidney protection of Cur administration.

## 2. Materials and Methods

### 2.1. Cell Culture and Treatments

The human embryonic kidney (HEK-293) cells were purchased from the Stem Cell Bank (Shanghai Chinese Academy of Sciences). DMEM media (Biochrome, Berlin, Germany) with 10% fetal bovine serum (Sigma, St Louis, MO) and 1% penicillin/streptomycin solution (Gibco-BRL, Grand Island, NY) were used for cell culture. A 37°C humidified atmosphere of 5% CO_2_ and 95% air was used for incubation condition.

The HEK-293 cells were grouped into seven: normal-glucose group (NG, 5.6 mmol/l glucose), high-glucose group (HG, 25 mmol/l glucose), glucose swing group (GF, oscillated glucose between 5.6 and 25 mmol/l every 8 h), GF+low-dose Cur group (GF+C20, cells managed with GF under 20 *μ*mol/l Cur treatment), GF+moderate-dose Cur group (GF+C40, cells managed with GF under 40 *μ*mol/l Cur treatment), GF+high-dose Cur group (GF+C80, cells managed with GF under 80 *μ*mol/l Cur treatment), and mannitol group (MG, 5.6 mM glucose plus 19.4 mM mannitol used for an osmotic pressure control).

### 2.2. Cell Transfections

HEK-293 cells with 70-80% density were seeded 24 h before transection. Then, the miR-489 inhibitor, LDHA overexpression plasmid, and their negative controls were transfected via the Lipofectamine 3000 reagent (Invitrogen, Carlsbad, CA, USA) into the cells for 48 h. Vectors of LDHA overexpression and control were acquired from http://Origene.com/. At 48 hours post transfection, cells were further analyzed in downstream experiments. Transfection was performed in triplicate.

### 2.3. Cell Counting Kit-8 (CCK-8)

The 5 × 10^3^ per well of HEK-392 cells were plated in 96-well plates. After 24 h serum starvation, cells were managed by different treatments. Then, 10 *μ*l CCK-8 solution (Kumamoto, Japan) was added in each well for 1 h incubation. The 450 nm optical density was recorded, and the mean value was calculated via three replicates.

### 2.4. Measurements of Lactate Acid and pH in Cell Supernatant

The Lac Colorimetric/Fluorometric Assay Kit (Jiancheng Biotech, CHN) and pH instrument (OHAUS STARTER 2C, USA) were used for lactate acid (lac) and pH value tests. All the steps were according to the instructions of manufacturer.

### 2.5. Measurement of PFK Activity

The Colorimetric Assay Kit was used for tests of the phosphofructokinase (PFK) activity (Sigma-Aldrich, USA). Managed HEK-293 cells were added with PFK assay solutions according to the instructions of manufacturer. The optical density value of the mixtures was recorded at every 30 s. 1.0 *μ*M per minute of NADH generation was mediated by one unit PFK. A NADH standard line was built to calculate the PFK activity. After normalization, the PFK activity was presented as milliunits/mg of protein.

### 2.6. Inflammation Marker

Contents of tumor necrosis factor alpha (TNF-*α*) and interleukin-1*β* (IL-1*β*) in culture supernatants were quantified by ELISA kits according to the instructions of the manufacturer.

### 2.7. Measurements of Oxidative Stress Markers

The activity of superoxide dismutase (SOD) and contents of malondialdehyde (MDA) were selected as oxidative stress markers, which are measured by the assay kits according to the instruction of the manufacturer. Activities of mitochondrial and cytoplasmic SOD were tested as previously described [46].

### 2.8. Annexin V-APC/7-AAD Double Staining

After different managements, HEK-293 cells were stained by the apoptosis assay kit of Annexin V APC/7-AAD cell (Beijing Bjbalb., CHN) according to the instructions of the manufacturer. Four subpopulations were evaluated: cells of Annexin V-APC-/7-AAD- are normal ones, cells of Annexin V-APC-/7-AAD+ are necrotic ones, cells of Annexin V-APC+/7-AAD- are early apoptotic ones, and cells of Annexin V-APC+/7-AAD+ are late apoptotic ones. The total rate of early plus late apoptotic cells was considered the apoptosis index.

### 2.9. RT-qPCR

Total RNA was extracted by the TRIzol reagent (Invitrogen, CA), and PCR amplification was used via the SYBR Green PCR kit (Thermo). Each sample was tested with three repetitive wells. GAPDH was used as internal reference. The 2^−ΔΔCt^ method was used for calculation. Primer sequences are shown in Table 1.

### 2.10. Western Blot

Extraction of total protein was performed via the method of RIPA lysis. The BCA method was applied to test protein concentration. The 5% fat-free milk-blocked membranes were incubated with anti-PKM2 (1 : 1000, Boaosen, bs-0102 M), anti-p-PKM2 (1 : 1000, CST, 3827), and anti-LDHA (1 : 2000, Boaosen, bs-34202R), respectively. The ratio of gray value between target protein and GAPDH was recorded.

### 2.11. Statistical Analysis

Data were presented as the mean value ± standard error of the mean (SEM). One-way ANOVA was used to calculate the differences among groups via the SPSS 22.0 software. A value of *p* < 0.05 was regarded as statistically different. All tests were technically repeated three times.

## 3. Results

### 3.1. Cur Protected HEK-293 Cell from GF-Triggered Cytotoxicity

**Figure 1:**
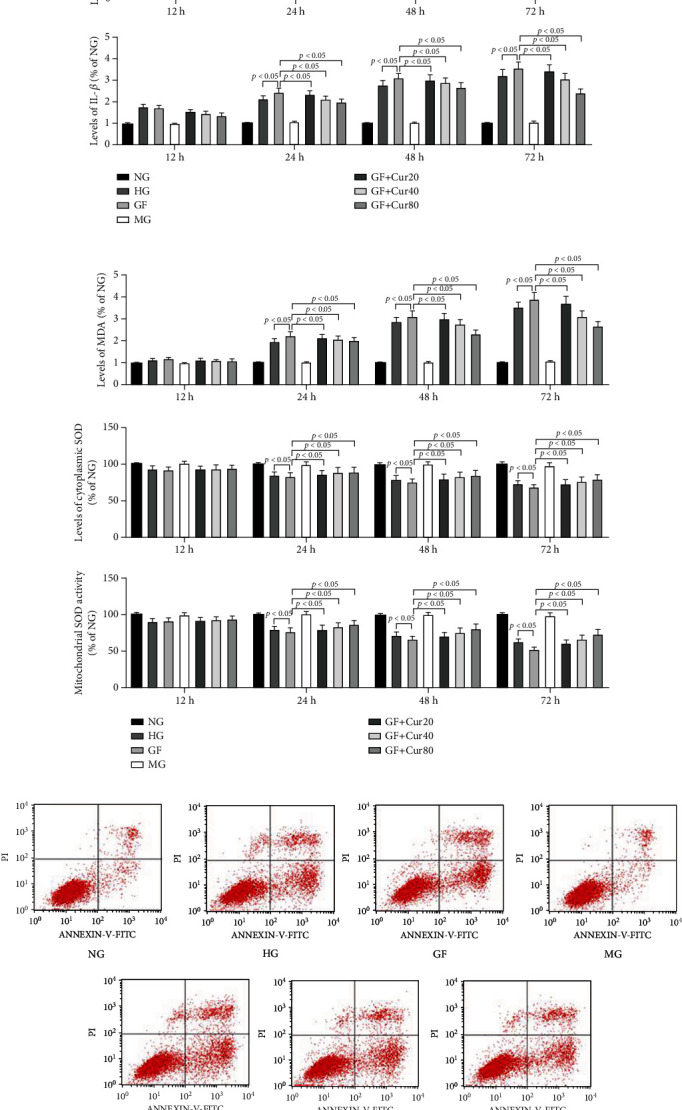
Cur protected HEK-293 cell from GF-induced cytotoxicity. (a) Viability of HEK-293 cells was measured by CCK8 at 24 h, 48 h, and 72 h. (b) Inflammatory markers of TNF-*α* and IL-1*β* at 24 h, 48 h, and 72 h. (c) Levels of oxidative stress marker, including MDA and mitochondrial and cytoplasmic SOD at 24 h, 48 h, and 72 h. (d) Apoptosis index was tested by flow cytometry at 48 h. GF deteriorated inflammation injury, oxidative stress, and apoptosis in HEK-293 cell, while Cur could alleviate this GF-induced cytotoxicity. Each error bar reflects the SEM of at least three independent sets.

First, we tested the levels of cytotoxicity triggered by different high-glucose conditions in the HEK-293 cell model and evaluated the protective effects of different doses of Cur. As shown in Figure 1, the cell viability of HEK-293 was markedly reduced in the GF group compared with NG and HG groups at 24 h, 48 h, and 72 h (Figure 1(a)). Compared with NG and HG, GF could obviously aggravate inflammation injury (TNF-*α* and IL-1*β*) (Figure 1(b)) and oxidative stress (MDA, mitochondrial and cytoplasmic SOD) (Figure 1(c)) at 24 h, 48 h, and 72 h in HEK-293 cells. The flow cytometry test indicated that the apoptosis index was dramatically exacerbated in the GF group compared with the NG and HG groups at 48 h, respectively (Figure 1(d)). Cur could significantly decrease inflammation damage under GF condition. Cur could prevent GF-triggered cytotoxicity by increasing the HEK-293 cell viability, reducing the inflammation injury and oxidative stress, and decreasing the apoptosis cell number in a dose-dependent manner. Totally, these results showed that GF aggravated inflammation injury, oxidative stress, and apoptosis in HEK-293 cell, while Cur could relieve GF's cytotoxicity.

### 3.2. Cur Prevented the GF-Induced Warburg Effect in HEK-293 Cells

**Figure 2:**
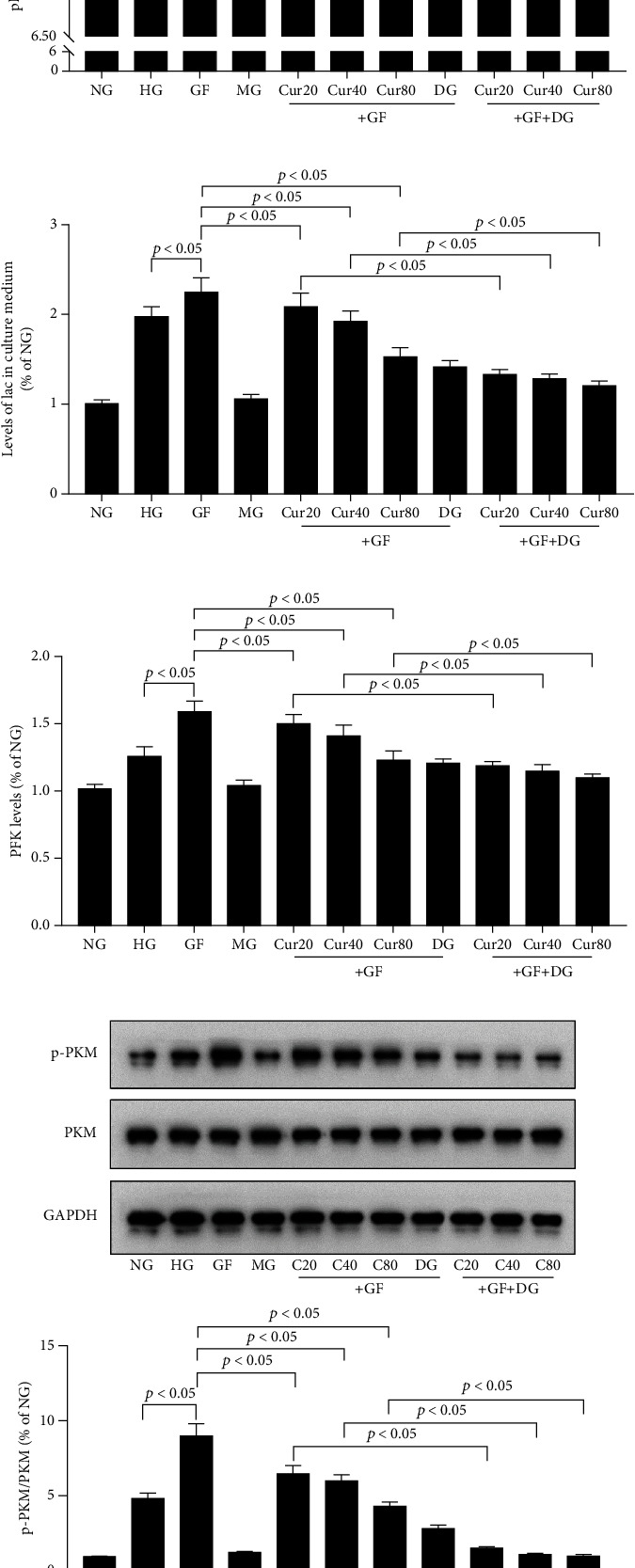
Cur prevented GF-induced aerobic glycolysis in HEK-293 cells. (a) pH in each group. (b) lac in each group. (c) PFK activity measured by colorimetric assay. (d) Western blot presenting PKM2 phosphorylation levels in each group. GF intensified aerobic glycolysis switch in HEK-293 cells, and Cur could weaken this intensification in a dose-dependent manner. Each error bar reflects the SEM of at least three independent sets.

Then, we tested the levels of the Warburg effect under different high-glucose circumstances and verified Cur's protective effects in the cell model of HEK-293. As shown in Figure 2, GF could obviously lead to aberrant cellular levels of energy metabolic product, such as the decrease of pH (Figure 2(a)) and increase of lac in a HEK-293 cell culture medium (Figure 2(b)). Our findings suggested that GF could enhance PFK activity in the HEK-293 cell model (Figure 2(c)). Western blot presented that p-PKM2/PKM2 was notably increased under the GF condition in the HEK-293 cell model (Figure 2(d)). As expected, these GF-induced energy switches could be dose-dependently prevented by Cur and 2-deoxyglucose (2-DG), a glycolytic pathway inhibitor. Taken together, these findings showed that GF deteriorated the Warburg effect in HEK-293 cells and Cur dose-dependently weakened these intensifications.

### 3.3. Cur Treatment on Different ncRNAs Related with Aerobic Glycolysis

**Figure 3:**
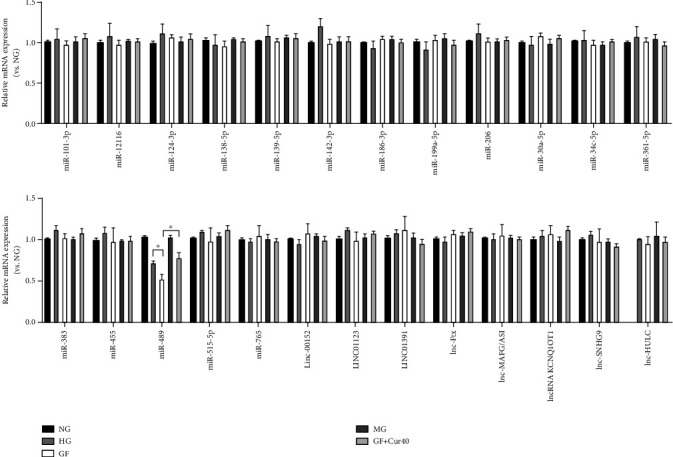
Effects of Cur on different ncRNAs related with the Warburg effect. We built a panel of 17 miRNAs and 8 lncRNAs that were involved in mediation of the Warburg effect. RT-qPCR results indicated that GF reduced the miR-489 content in the HEK-293 cell model, and a moderate dose of Cur (40 *μ*M) could prevent this downregulation. Each error bar reflects the SEM of at least three independent sets.

Next, we built a panel of 17 miRNAs and 8 lncRNAs that were involved in mediation of Warburg effect. As shown in Figure 3, RT-qPCR results indicated that GF reduced the miR-489 content in the HEK-293 cell model and a moderate dose of Cur (40 *μ*M) could prevent this downregulation. Therefore, we selected miR-489 as the entry point for further experiments.

### 3.4. Cur Treatment Prevented GF-Triggered Aerobic Glycolysis by Regulating miR-489 in HEK-293 Cells

**Figure 4:**
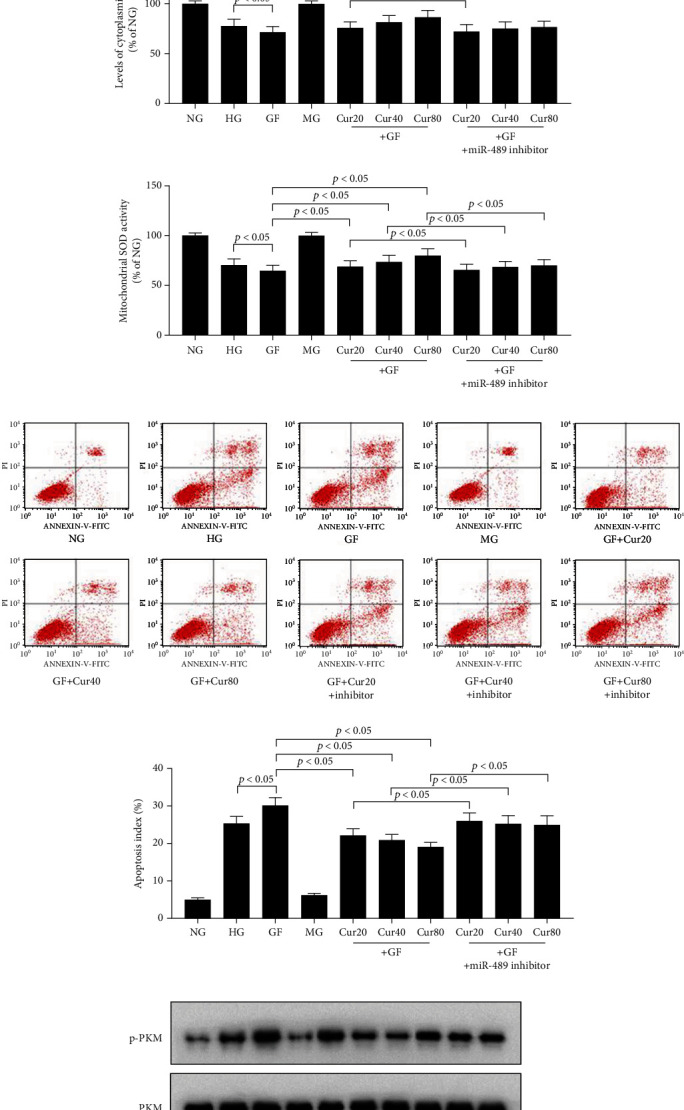
Cur prevented GF-triggered aerobic glycolysis by regulating miR-489 in HEK-293 cells. (a) RT-qPCR results showed that Cur could alleviate GF-induced reduction of miR-489 in a dose-dependent manner. (b) Inhibition effects of miR-489 inhibitor tested by RT-qPCR. (c) Viability of HEK-293 cells was tested by CCK8 at 48 h. (d) TNF-*α* and IL-1*β* at 48 h. (e) Levels of oxidative stress marker, including MDA and mitochondrial and cytoplasmic SOD at 48 h. (f) Apoptosis index was tested by flow cytometry at 48 h. Inhibitor of miR-489 could reduce Cur's protective effects in the fields of cell viability, inflammation injury, and oxidative stress. (g, h) The suppression of aerobic glycolysis by Cur treatment was prevented by the miR-489 inhibitor in HEK-293 cells. Each error bar reflects the SEM of at least three independent sets.

In the following tests, we intended to evaluate the role of miR-489 in the Cur-triggered attenuation of the Warburg effect under the GF condition. First, we found Cur could alleviate GF-induced reduction of miR-489 in a dose-dependent manner in HEK-293 cells (Figure 4(a)). Then, we found that downregulation of miR-489 by inhibitor (Figure 4(b)) could weaken the protective effects of Cur in the fields of cell viability (Figure 4(c)), inflammation injury (Figure 4(d)), oxidative stress (Figure 4(e)), and apoptosis cell number (Figure 4(f)). The suppression of aerobic glycolysis by Cur treatment was also prevented by miR-489 inhibitor (Figures 4(g) and 4(h)) in HEK-293 cells. Taken together, these results presented that Cur prevented GF-triggered aerobic glycolysis by upregulating miR-489 in HEK-293 cells.

### 3.5. Cur Treatment Prevented GF-Triggered Aerobic Glycolysis via the miR-489/LDHA Axis in HEK-293 Cells

**Figure 5:**
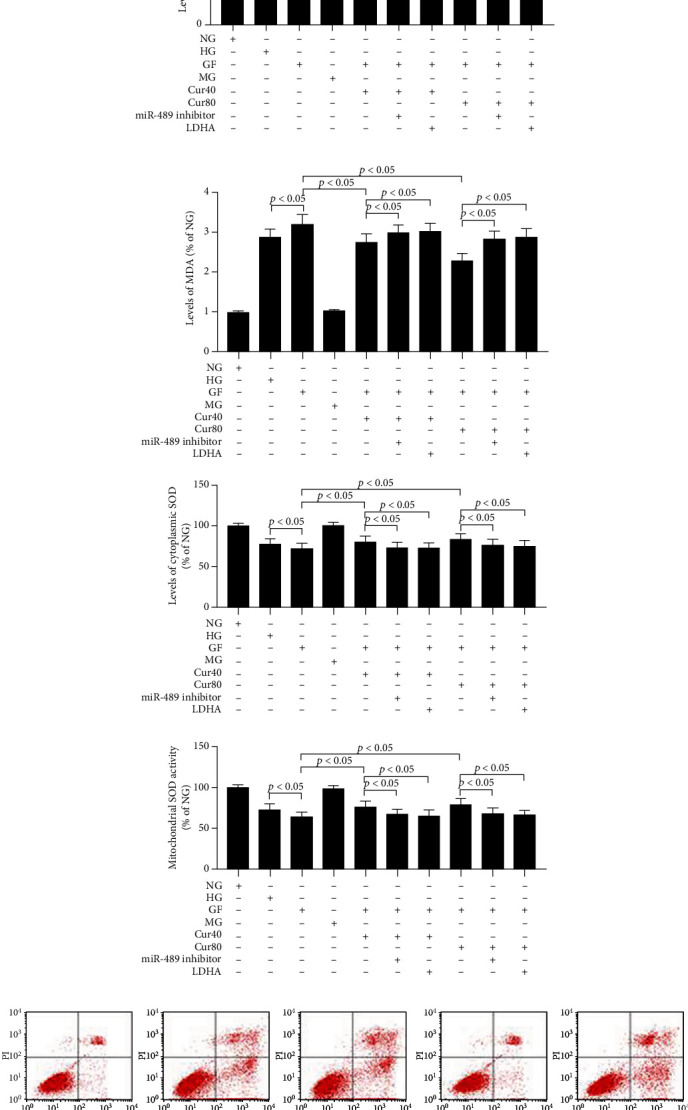
Cur prevented GF-triggered aerobic glycolysis via the miR-489/LDHA axis in HEK-293 cells. (a–c) RT-qPCR and WB results showed that Cur could alleviate GF-induced increase of LDHA in a dose-dependent manner at 48 h. (d) Viability of HEK-293 cells was tested by CCK8 at 48 h. (e) TNF-*α* and IL-1*β* at 48 h. (f) Levels of oxidative stress marker, including MDA and mitochondrial and cytoplasmic SOD at 48 h. (g) Apoptosis index was detected by flow cytometry at 48 h. Downregulation of miR-489 or upregulation of LDHA could weaken the protective effects of Cur in the fields of cell viability, inflammation injury, oxidative stress, and apoptosis. (h, i) The suppression of aerobic glycolysis by Cur treatment was prevented by the miR-489 inhibitor or LDHA pcDNA in HEK-293 cells. Each error bar reflects the SEM of at least three independent sets.

Next, we choose the miR-489/LDHA axis for further investigation. This axis has been reported as a new explanation for the Warburg effect [41]. First, we took note of the increase on LDHA mRNA (Figures 5(a) and 5(b)) and protein (Figure 5(c)) under the GF circumstance. These increases could be dose-dependently prevented by Cur administration. And these Cur's effects could be weakened by the miR-489 inhibitor. LDHA pcDNA or miR-489 inhibitor could also whittle Cur's protective effects including the improvement of cell viability (Figure 5(d)), decrease of inflammation damage (Figure 5(e)) and oxidative stress (Figure 5(f)), reduction of apoptosis cell number (Figure 5(g)), and weakening of aerobic glycolysis switch (Figures 5(h) and 5(i)) in the HEK-293 cell model. Therefore, these results suggested that Cur prevented GF-triggered aerobic glycolysis via the miR-489/LDHA axis in HEK-293 cells.

## 4. Discussion

In comparison to constant high- or low-glucose condition, oscillating levels of blood glucose can induce harder cytotoxicity, such as inflammatory injury, oxidative stress, and apoptosis in the fields of DN development [3, 10] and other DM-related complication [47]. In the present study, we used HEK-293 cell to build a cellular model exposed to different high-glucose circumstances which partly mimic constant and oscillating high-glucose conditions in DM patients. We found Cur treatment presented a reduction of aerobic glycolysis rate in HEK-293 cell under the GF condition. This metabolic shift is related to a reduction in inflammation injury, oxidative stress, and apoptosis against the simulated GF-related cytotoxicity. Our finding provided further evidence to support the kidney protection of Cur in the fields of cellular energy metabolism.

In this study, we first tested the levels of cytotoxicity and the Warburg effect triggered by different high-glucose conditions in HEK-293 cell model. Our results showed GF could deteriorate inflammation injury, oxidative stress, and apoptosis in HEK-293 cell. Previous studies have shown that GF condition had apoptosis-inducing and oxidative stress abilities on cells, including MCs [4], podocytes [5], and vascular endothelial cells [6]. Further, another study presented that GF increased aerobic glycolysis switch and aggravated renal injury, such as reducing cell proliferation and exacerbating inflammation and apoptosis, in a MC cell model [10]. Our results were in accordance with previous studies, and we testified the GF-related renal injury in HEK-293 cell which further verified the association between GF and DN.

Then, we found Cur treatment could prevent GF-triggered cytotoxicity in a dose-dependent manner. Cur is a bioactive component derived from the rhizome of turmeric, which is a classical herb and has been used for thousands of years in metabolic disease in China. Cur is involved in numerous crucial genetic and biochemical pathways and produces renal protective effects [48]. It is a potential therapeutic drug against DM diseases. A metastudy including 24 clinical trials indicated that the levels of glycosylated hemoglobin were obviously reduced in Cur-treated patients [49]. Cur was found to inhibit podocyte cell apoptosis, accelerating cell autophagy, alleviating podocyte EMT, and reducing inflammation injury in the in vivo and in vitro models of DN [13–16]. In our study, we found Cur could prevent the Warburg effect that enhanced under the GF condition, which might be a novel target in the understanding of Cur's kidney protection. Cur takes part in the mediation of aerobic glycolysis. However, these studies are most regarding to cancer cells [18, 19]. Our results presented the evidence that Cur also reduces aerobic glycolysis switch in HEK-293 cells.

The mechanism of the Warburg effect is still unclear. We built a panel of 17 miRNAs [26–29, 31–43] and 8 lncRNAs [29, 30, 32, 35, 36, 43–45] that were previously reported to be regulators of the Warburg effect. The results indicated that GF reduced the miR-489 content in the HEK-293 cell model and a moderate dose of Cur (40 *μ*M) could prevent this downregulation. Then, we performed rescue experiments to confirm that Cur prevents GF-triggered aerobic glycolysis via the miR-489/LDHA axis in HEK-293 cells. The axis of miR-489/LDHA has been reported as a new explanation for the Warburg effect. miR-489 was reported to inhibit the growth of multiple myeloma by regulating the LDHA-mediated glycolytic metabolism [41]. Inhibition of miR-489 obviously decreased atherosclerotic lesion in a renal injury mouse model [50]. LDHA plays as a rate-limiting enzyme and catalyzes pyruvate into lactate instead of leading into the tricarboxylic acid cycle under the aerobic glycolysis condition. Pancreatic islets of DM individuals showed an increase in the LDHA expression [51]. The downregulation of LDHA could significantly improve DM's glucose metabolism [52]. Inhibition of LDHA decreased reactive oxygen species production, reduced lactate secretion, and rescued beta-cell apoptosis [53]. Our results showed that reduced expression of miR-489 in GF group could increase the apoptosis index as determined by flow cytometry in HEK-293 cell. This effect of miR-489 was also found in a LPS-induced cell injury model of human embryonic lung WI-38 cells [54]. Interestingly, some studies presented that upregulation of miR-489 could facilitate apoptosis in tumor cells, such as human pancreatic cancer PANC-1 cells [55] and human hepatocellular carcinoma cells [56]. These different effects of miR-489 may be due to particular characteristics of tumor cells.

There were some limitations in our study. We only built a panel of the Warburg effect-related miRNAs and lncRNAs, but not involving DN- or GF-associated ncRNAs. It was not testified that whether higher expression of LDHA or lower level of miR-489 was responsible for renal damage. In vivo evidences were required to prove adverse effects of GF to aerobic glycolysis on DN development. These will be performed in a future study.

In conclusion, our study indicated that Cur prevented GF-triggered renal injury by inhibition of aerobic glycolysis via the miR-489/LDHA axis in the HEK-293 cell model. Although further follow-up experiments are required in the future, our results may provide in-depth understanding on the mechanism of GF-triggered renal injury and Cur's protective capacity.

## Figures and Tables

**Table 1:** Primer sequences.

	Forward	Reverse
miR-101-3p	5′-GCCGCCACCATGGTGAGCAAGG-3′	5′-AATTGAAAAAAGTGATTTAATTT-3′
miR-12116	5′-GCCTTTGGTTCTTCTTAG-3′	5′-GCTCTGGGTTCTTCTTAG-3′
miR-124-3p	5′-CGGCAAGTTGTCGGAGACG-3′	5′-CCTGGAGGTTGGGATGCTCT-3′
miR-138-5p	5′-GCTTAAGGCACGCGG-3′	5′-GTGCAGGGTCCGAGG-3′
miR-139-5p	5′-TCTACAGTGCACGTGTC-3′	5′-GAATACCTCGGACCCTGC-3′
miR-142-3p	5′-GGCCCATAAAGTAGAAAGC-3′	5′-TTTGGCACTAGCACATT-3′
miR-186-3p	5′-CGCGCAAAGAATTCTCCTTT-3′	5′-AGTGCAGGGTCCGAGGTATT-3′
miR-199a-5p	5′-TCAAGAGCAATAACGAAAAATGT-3′	5′-GCTGTCAACGATACGCTACGT-3′
miR-206	5-GCGTCTGGAATGTAAGGAAGTG-3′	5′-GTGCAGGGTCCGAGGT-3′
miR-30a-5p	5′-AACGAGACGACGACAGAC-3′	5′-TGTAAACATCCTCGACTGGAAG-3′
miR-34c-5p	5′-GCG CAT CCC TTG CAT GGT-3′	5′-AGT GCA GGGTCCGAG GTATT-3′
miR-361-5p	5′-GCCGAGTTATCAGAATCTCCA-3′	5′-CTCAACTGGTGTCGTGGA-3′
miR-383	5′-GACAGACCTTGTGAAGGTGACTCTG-3′	5′-GACCAGCTTCCAGAGGACAAGATCTC-3′
miR-455	5′-TAAGACGTCCATGGGCAT-3′	5′-GTGCAGGGTCCGAGGT-3′
miR-489	5′-CCCCGCCGTGACATCACATAT-3′	5′-CCAGTCGGTGGCTGCCGTATA-3′
miR-515-5p	5′-TTCTCCAAAAGAAAGCACTTTCTG-3′	5′-CTCGCTTCGGCAGCACA-3′
miR-765	5′-GUAGCCAAGGAATCCGAAGGA-3′	5′-GCGAGGAAGGAGGAGGAAGGT-3′
LINC00152	5′-CTCCAGCACCTCTACCTGTTG-3′	5′-GGACAAGGGATTAAGACACACA-3′
LINC01123	5′-ACAGTGGCCGCACGCATAGCTG-3′	5′-CTGACGACCGAGGTGACAACGATGA-3′
LINC01391	5′-TGGCACCCGCTATGTCCA-3′	5′-GTAGCAGGGATTCTGTCTG-3
lnc-Ftx	5′-GAATGTCCTTGTGAGGCAGTTG-3′	5′-TGGTCACTCACATGGATGATCTG-3′
lnc-MAFG/ASI	5′-ATGACGACCCCCAATAAAGGA-3′	5′-CACCGACATGGTTACCAGC-3′
lncRNA KCNQ1OT1	5′-TTGGTAGGATTTTGTTGAGG-3′	5′-CAACCTTCCCCTACTACC-3′
lnc-SNHG9	5′-CCCGAAGAGTGGCTATAAACG-3′	5′-GGAGGACCAGTGTCCTAAGTGAA-3′
lnc-HULC	5′-CTGGCAATAAACTAAGCA-3′	5′-CAACATAATTCAGGGAGAA-3′
LDHA	5′-CTTCCTCAGTGTCCCATGTATC-3′	5′-TTTCCCCACACCATCTCAAC-3′
GAPDH	5′-TGCACCACCAACTGCTTAGC-3′	5′-GGCATGGACTGTGGTCATGAG-3′

## Data Availability

The datasets used and/or analyzed during the current study available from the corresponding authors on reasonable request.
